# Team Emotional Intelligence: Emotional Processes as a Link Between Managers and Workers

**DOI:** 10.3389/fpsyg.2021.619999

**Published:** 2021-03-24

**Authors:** Rosa Mindeguia, Aitor Aritzeta, Alaine Garmendia, Edurne Martinez-Moreno, Unai Elorza, Goretti Soroa

**Affiliations:** ^1^Department of Basic Psychological Processes and Their Development, Faculty of Psychology, University of the Basque Country, Donostia-San Sebastian, Spain; ^2^Mechanical and Industrial Production Department, Faculty of Engineering, University of Mondragón, Mondragón, Spain; ^3^Department of Social Psychology and Methodology of Behavioral Sciences, Faculty of Psychology, University of the Basque Country, Donostia-San Sebastian, Spain; ^4^Department of Clinical and Health Psychology and Research Methodology, Faculty of Psychology, University of the Basque Country, Donostia-San Sebastian, Spain

**Keywords:** team emotional intelligence, leadership, positive emotions, cohesion, passion

## Abstract

Research has shown that transformational leaders are able, through emotional contagion mechanisms, to transmit their emotions and boost positive feelings among their followers. Although research on leadership and team processes have shown a positive relation between transformational leadership and workers' well-being, there is a lack of studies examining the “black box” of this association. The present study aimed to assess the mediation effect of team emotional intelligence (TEI) of the management team on the relationship between management's transformational behaviors and employees' responses. Data were gathered from two sources: 1,566 managers grouped into 188 teams pertaining to a total of 90 firms, and 4,564 workers from the same 90 firms. The results showed that management team TEI and the emotional state of “passion” among employees had a full mediation effect on the relationship between management teams' transformational leadership and employees' cohesion. Implications of these results are discussed.

## Introduction

Organizational scholars have long been interested in well-being at work and the associated positive attitudes and experiences of leaders and employees. Therefore, the literature on the antecedents and consequences of happiness and well-being at work is rapidly developing (Fisher, 2010). Regarding antecedents, the role of leadership seems especially relevant (García-Buades et al., 2020); leadership is defined as a process of social influence through which a leader influences subordinates' feelings, perceptions, and behaviors (Pirola-Merlo et al., 2002). Some investigations in this field have shown that leaders improve employees' performance and motivate them to make achievements beyond the leader's expectations and organizational obligations (Edú-Valsania et al., 2016).

Although recent meta-analytic studies have linked leadership style to performance at different levels of analysis, that is to say, the individual, the team, and the organization (e.g., Tseng and Levy, 2018), knowledge is still lacking regarding the effect of leadership style at the organizational level. Although few studies have explored the effect of leadership style at organizational level, transformational leadership has been identified as an effective behavior related to a different organizational and to work-unit outcomes and employee well-being (for a review see, García-Buades et al., 2020). However, one meta-analysis of research in this field showed that there are important potential mediators of the outcomes of transformational leadership that need to be examined (Wang et al., 2011), with the individual and group affective dimensions being important sources of variability.

Overall, these studies highlight the need for further investigation of leadership styles and affective dimensions from a multilevel perspective. Multilevel research has demonstrated that a given variable examined at the individual level is often not comparable to the same variable at a higher level of analysis (Ashkanasy, 2003; Ashkanasy and Dorris, 2017). Ashkanasy (2003) proposed the multilevel model of emotion in organizations and stated that studying emotional processes only at the individual level could lead to an incomplete understanding of how different variables may influence performance. The present study aims to contribute to this growing area of research by considering both the effect of management teams' leadership style on organizations (Roh et al., 2019) and the potential mediation effects of emotional dimensions located at different levels of analysis.

The ubiquity of emotion in teams, and its influence on team processes, is widely acknowledged (Menges and Kilduff, 2015). For example, it has been shown that, shared positive moods through work units might influence their team's motivational (e.g., team goal commitment), attitudinal (e.g., team satisfaction), and behavioral (e.g., proactive behaviors) processes (García-Buades et al., 2020). One of the emotional constructs that has been identified as an influence source of variability in different variables related to group behavior is team emotional intelligence (TEI). Druskat and Wolff (2001) define TEI as ‘*the ability of a group to develop a set of norms that manage emotional processes*’ (Druskat and Wolff, 2001, p. 133). This set of norms or expected behaviors is generated through subjective emotional experiences that group members share, and it will define their subsequent emotional experiences (Wolff et al., 2006).

In this research, we used the definition given by Aritzeta et al. (2020, p. 2) for TEI, who defined it as “the ability of a team to pay attention to the feelings of teammates, to understand the emotions felt in the team, and to use positive thinking to repair negative moods in the team.” Therefore, it must be mentioned that TEI, in our case, is not synonymous with the aggregated emotional intelligence of individual team members. Rather, it refers to the ability generated by the team as a whole to pay attention to, to be clear about, and to regulate the emotions felt within the team. This definition of TEI is based on the theoretical model initially proposed by Mayer and Salovey (1997), and, in the field of work and organizational psychology, has been one of the most widely-used models for measuring individual perceived EI (for a review see: Kotsou et al., 2018). In the article published by Aritzeta et al. (2020) they measured TEI using a “team reference model” and not aggregating individual responses. In their process of creating the TEI measure (the T-TMMS described below), they used the “consensus-based change-of reference” strategy, following Chan's (1998) theory of group-level composition models. This strategy supports the idea that a group-level characteristic can be examined by changing the reference from the individual to the group level; that is to say, by changing the framework of the tapped characteristic from the individual to the group level. Additionally, the within-group agreement should be ensured by means of the James intercoder reliability index (James et al., 1993). The reference framework for responding to items was changed from the individual self-evaluation (e.g., “I pay a great deal of attention to my feelings”) to the perception of team/group experience (e.g., “In this team, we are able to describe our feelings”). Thus, it is a direct group level measure that measures the degree to which, on average, leaders or workers belonging to a stable team perceive that their team attends to feelings and values them, is clear, rather than confused, about feelings, and adopts positive thinking to repair negative group moods.

If we consider the team to be an entity in itself, then its performance depends less on the individual characteristics of team members than on the structures and patterns of behavior they generate within a specific team (Ashkanasy, 2003; Elfenbein, 2006), in other words, on the TEI. To put it another way, each team has a singular nature that derives from the experiences, learning, norms, and ways of functioning that define it; this idiosyncratic quality of teams may be defined in terms of team-level variables such as emotional climate (Peñalver et al., 2017) and TEI (Lee and Wong, 2019).

Within the literature on TEI, various theories have been used in an attempt to explain how group emotional processes may affect individuals. Based on the notion of emotional contagion (Barsade, 2002) and affective events theory (AET; Weiss and Cropanzano, 1996; Weiss, 2002) it has been proposed that the extent to which teams engage in interpersonal emotional processes could influence not only a team's effectiveness but also — through trickle-down effects — employees' individual affect and behaviors (Tse et al., 2018). Although team emotional intelligence can be expected to influence intrateam conflict (i.e., task conflict and relationship conflict) and team effectiveness (i.e., team performance, innovation, and cohesion), team emotional intelligence has been largely unexplored (Lee and Wong, 2019).

The results of the review by Diener et al. (2020) show that positive emotions influence key variables within organizations, leading, for instance, to increased creativity, commitment, and effectiveness, not only of the team but also of its members (Diener et al., 2020). A positive emotional climate within teams has been associated with a more positive view of the future (George, 2011) and better group cohesion, since members feel a stronger commitment to the group's objectives (Peñalver et al., 2017). Cohesion is a multidimensional construct consisting of interpersonal attraction, commitment to task, and group pride that keeps members together (Mullen and Copper, 1994). Social resources, such as cohesion, promote socially-integrated groups that are coordinated and committed to group goals (Beal et al., 2003).

**Figure 1:**
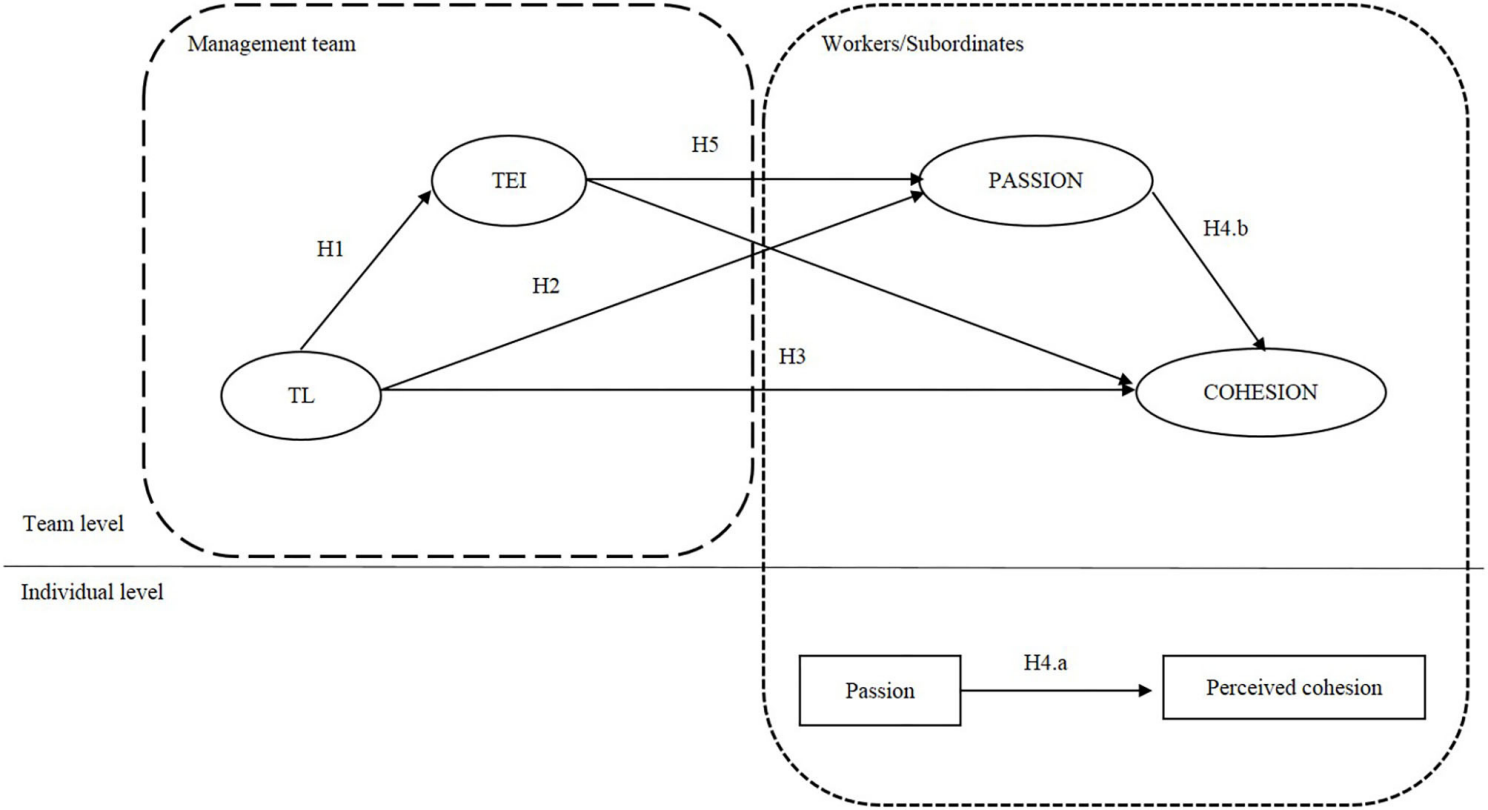
Proposed model. TL, Transformational leadership; TEI, Team emotional intelligence; H1-H5, Hypotheses 1-5.

In light of the above, the present paper analyzes the mediation effect of the management team's TEI on the relationship between that team's transformational behaviors and employees' positive emotions and cohesion. Our goal in doing so is to respond to calls for a multilevel study of EI and to develop a research model that simultaneously analyzes the multilevel influence of TEI (Ashkanasy, 2003; Troth et al., 2017) and leader-member exchange (Tse et al., 2018). Our proposed model is shown in Figure 1.

## Hypothesis Development

In this section we will describe the theoretical development of the hypothesized model shown in Figure 1. We begin by considering intragroup processes, such as the relationship between transformational leadership and TEI in managerial groups, and then consider the relationship between management teams and followers by analyzing the mediation effect of positive emotions and TEI.

### Transformational Leadership and TEI

Transformational leadership is one of the most widely studied leadership styles in the field of organizational research (Avolio et al., 2009). At the organizational level, it has been shown that transformational leaders influence organizational performance by means of their direct leadership of the top management team. More specifically, by increasing team cohesion, motivation, and goal congruence within the top management team, transformational leaders increase the levels of organizational performance (Colbert et al., 2008).

This leadership style is based on four primary behaviors: inspirational motivation, idealized influence, intellectual stimulation, and individualized consideration (Bass, 1985). In other words, transformational leaders are able to: (1) project a charismatic vision that is believed in by group members, (2) inspire group members to perform above normal standards, (3) provide intellectual stimulation for group members, and (4) look after the emotional needs of group members. Tse et al. (2008) found that leaders help to the quality of team members' exchanges, and that this process was facilitated in teams defined by a positive affective climate.

Some studies have shown a relationship between transformational leadership and emotional intelligence mediating the association between emotional intelligence and counterproductive work, and the relationship between emotional intelligence and organizational commitment (Foster and Roche, 2014; Hussein and Yesiltas, 2020). Also, a recent research work showed that leaders' transformational leadership had a significant effect on employee engagement for the mediating role of emotional intelligence (Milhem et al., 2019).

At the team level, the relationship between transformational leadership and TEI has been demonstrated in a recent study by Lopez-Zafra et al. (2017). Accordingly, when leaders influence the processes, behaviors, norms, and climate within work teams, their individual personality may influence the emerging climate of the team (Stubbs and Wolff, 2008) as well as the ability of the team to manage their emotional states (Aritzeta et al., 2020). Being part of a work team implies a complex combination of information processing and emotional responding that could influence team members' responses, as the same worker may experience different emotional responses to a dramatic event on two different teams, depending, for example, on that team member's leadership style and how it influences individual perceptions of TEI (Ghuman, 2016).

As transformational leaders care about their followers and appeal to them on an emotional level, followers “*have many opportunities to reinforce (vs. douse) each other's commitment to their common cause”* through a process of social influence and emotional contagion (Hatfield et al., 1994; Klein and House, 1995; 192; see also Barsade, 2002). Consequently, transformational leadership behaviors help to generate emotionally competent norms, leading to higher TEI (Lopez-Zafra et al., 2017), influencing the way in which teams members perceive the ability of the team to manage emotions.

Based on the above we hypothesize that:

H1: Transformational leadership of the management team is positively related to its TEI.

### The Mediating Effect of TEI and Affect

Employee well-being can be defined as the as the overall quality of an employee's experience of work and performance. The literature in this regard presents three different approaches that refer to the subjective experiences of well-being, the health perspective of well-being and social well-being (Pagán-Castaño et al., 2020). In this research, we will analyze subjective (through passion emotional state) and social (through cohesion) well-being.

A great number of concepts may be construed as belonging to the well-being construct, including job satisfaction, job involvement, affect, organizational commitment, work engagement, cohesion, positive and negative emotions and moods at work (Fisher, 2014).

Carron and Brawley (2000) define cohesion as “*a dynamic process that is reflected in the tendency for a group to stick together and remain united in the pursuit of its instrumental objectives and/or for the satisfaction of member affective needs*.” (2000: 213).

In fact, leadership and team researchers have demonstrated a positive relation between transformational leadership and cohesion (Lim and Ployhart, 2004; Schaubroeck et al., 2007; Colbert et al., 2008), although it has also been pointed out that there are a number of processes which may mediate this relationship (García-Morales et al., 2008).

One issue that has generated growing interest among researchers in this field is the idea of “trickle-down effects,” whereby the perceptions, feelings, attitudes or behaviors of a manager influence the perceptions, feelings, attitudes or behaviors of a supervisor, which in turn influence the perceptions, feelings, attitudes or behaviors of subordinates (e.g., Wo et al., 2019). From this perspective, transformational leadership behaviors may trickle down the organizational hierarchy from leaders to employees and influence employees' well-being (Dvir et al., 2002; Yang et al., 2010).

Therefore, we hypothesize that:

H3: Transformational leadership by the management team will be positively related to employee's group cohesion.

The literature on trickle effects has focused predominantly on cognition-based constructs such as leadership (Mayer et al., 2009) rather than on affective constructs, even though the latter might also have an important effect on the relationship between managers and subordinates. Indeed, recent research suggests that strong emotions may be more likely to be transmitted across different levels of an organizational hierarchy, and thus, in comparison with more cognitive aspects, they would exert more influence on members of the organization (Wo et al., 2019).

In this context the affective events theory (AET; Weiss and Cropanzano, 1996) is a well-known framework used for understanding the emotional relationships between leadership behaviors and team results (Gooty et al., 2010). According to the AET, leaders create affective events which have a positive or negative influence on teams, shaping the intensity and form of their emotional responses, in other words, their emotional state. Many investigations have recognized that leaders are able to increase positive feelings in their followers (George, 2000; Dasborough and Ashkanasy, 2002), which, in turn, affect their work attitudes and behaviors (McColl-Kennedy and Anderson, 2002; Gooty et al., 2010). Within circumflex models of emotions (Russell, 1980; Bruch and Ghoshal, 2003), these strong positive emotions, such as joy and pride, compose the emotional state referred to as “passion.”

A number of review articles have highlighted the need to separate the effect of positive and negative emotions so as to examine each of them more clearly (Ashkanasy and Dorris, 2017; Diener et al., 2020). In this respect, it is worth noting that circumflex models of emotions (Russell, 1980) have proved to be useful for explaining the relationship between leadership, affect, and emotions (Van Knippenberg et al., 2008). These models understand that emotions such as anger, sadness, and fear share a common set of basic psychological properties that are defined by two dimensions: quality (pleasure vs. displeasure) and activation (high or low activation). The intersection of quality and activation determines the affective state, which can be referred to as, for example, comfort (pleasure and low activation), resignation (displeasure and low activation), passion (pleasure and high activation) or aggression (displeasure and high activation). Generally speaking, discrete emotions are used to generate one of these dimensions. Drawing on this perspective, and given that research shows that leaders are capable of generating strong positive emotions in their followers (Dasborough and Ashkanasy, 2002), the present study focuses on the emotional state referred to as “passion,” which is composed of four discrete emotions, each of which is characterized by a combination of pleasure and high activation: enthusiasm, pride, joy, and excitement.

The review published by (Diener et al., 2020), identified these positive emotions to produce positive changes in cognitions, behavior, affect, and physiology that lead to positive personal and social outcomes. The longitudinal study conducted by Casper et al. (2019) showed that individually, positive affect at work predicts an increase in positive interpersonal work events. In other words, at an individual level, employees who experience positive affect at work might perceive interactions with co-workers in a more positive way and, thus, perceive more cohesion.

We earlier mentioned the notion of emotional contagion, which refers to the processes whereby moods and emotions are transferred from one individual to other individuals (Kelly and Barsade, 2001). If we assume this logic to the team level, moods shared by team members might also affect their team's motivational, attitudinal, and behavioral processes over specific periods of time (Kelly and Spoor, 2007; George, 2011). Shared positive feelings generally promote social integration and, indirectly, enhance task performance (Knight and Eisenkraft, 2015). Moreover, when members collectively experience positive feelings in team meetings, these pleasant emotions push them to consider pursuing and valuating the importance of team goals, helping them to feel more committed to these goals (Seo et al., 2004) and, therefore, enhancing cohesion.

Given the multilevel influence of emotions and the structure of organizations (Ashkanasy, 2003), the model we propose in this paper aims to analyze the aforementioned variables from the multilevel perspective. More specifically, we seek to consider the individual variability between workers' emotional state of passion and the perception of cohesion that each worker has. In this respect, we hypothesize that:

H2: Transformational leadership by the management team will be positively related to the employee's passion.
H4: Employee passion will mediate the relationship between Transformational leadership and cohesion.
H4b: Group-level passion will be positively related to group cohesion.
H4a: Passion will be positively related to the individual perception of cohesion.

Research suggests that TEI may be a key construct that facilitates a leader's adaptive behavior. Teams with high TEI acquire better organizational understanding, leading to better emotional management not only inside the teams but also when the group deals with individuals and groups beyond the group's boundary (Stubbs and Wolff, 2008). These teams are likely to recognize and respect the emotional expressions of followers (e.g., George, 2000), and they also respond better to their emotions (Chang et al., 2011) and use this information to activate employees' emotion (George, 2000).

Finally, Ashkanasy and Dorris (2017) mentioned that a leader's behavior toward subordinates is observed in team-member relationships, which, in turn, reflect the leader's performance via processes like emotional contagion. Such processes lead to an organizational management response to the leaders. Teams with high TEI take on the role of “emotion manager” in order to establish a positive “affective tone,” both for their subordinates' benefit and so as to create positive affective events for them (Pescosolido, 2002).

Based on the above, we hypothesize that the relationship between a management team's transformational leadership, passion, and cohesion will be mediated by TEI through two processes: (1) by developing a better understanding of the team and improving the ways in which leaders respond to followers' needs; and (2) through the emotional contagion of positive emotions and trickle-down effects.

H5: TEI will be positively related to passion at the group level and will mediate the relationship between a management team's transformational leadership and passion.

## Method

### Participants

Data for this study were gathered between 2014 and 2016 from two sources: 1,566 managers grouped into 188 teams pertaining to a total of 90 firms, and 4,564 workers from the same 90 firms, all of which are part of the same corporation in Spain. Each leader team (between 4 and 9 members) manages each work unit that the workers come from. These teams work together every day, making strategical decisions for the organization and managing their work units. The time lag between leaders and employee responses was 1 week.

The data from the 1,566 managers were used only at team level (since we analyze them as a team); therefore, the final sample size for the model was 4,564 workers at the individual level and 188 work units and leader teams at the group level.

The Corporation is distributed across different economic sectors: Industry (*N* = 30; 33.3%), the service sector (*N* = 22; 24.4%), education (*N* = 7; 7.8%), and distribution (*N* = 31; 34.4%). In terms of size, 47.8% (*N* = 43) are small organizations (> 50 workers), 40% (*N* = 36) are medium-size organizations (between 50 and 200 workers), and 12.2% (*N* = 11) are large organizations (more than 200 workers). In the total sample, 38% of participants were female, and the average age was 42 years (SD = 8.68).

### Procedure

Prior to collecting any data, we sought permission from the top managers of all the organizations and identified all the work units and manager teams (of each unit) participating in the study. Manager teams answered the questionnaire 1 week before workers did.

The questionnaires were distributed in two ways, with participants being randomly selected to respond either via email or using the paper-and-pencil method (hard copy). The paper-and-pencil administrations took place in large meeting rooms under the supervision of a human resources manager from the employees' firm. All responses (both email and hard copy) were anonymous and Spanish data protection law was complied with throughout. The study has the approval of the ethics committee. There were no differences in questionnaire responses related to the method of administration (online vs. paper-and-pencil).

The data obtained were incorporated into a file for statistical analysis using IBM SPSS 24 and Mplus 7. Data from leader teams were aggregated and merged with workers' data using the organizational work unit as the key variable.

### Measures

#### Individual-Level Measures

##### Passion

The dimension considered for this construct is derived from Russell's circumflex model of emotion classification (Russell, 1980). The “Passion” dimension (high intensity and pleasure) comprised four discrete emotions: enthusiasm, pride, joy, and excitement (e.g., “*In my work I usually feel enthusiastic”*). The Cronbach's alpha obtained in the present study was 0.85 [0.85, 0.86] and omega was 0.84 [0.86, 0.86].

##### Cohesion

Cohesion between workers was assessed using a scale adapted and validated previously by Aritzeta et al. (2020). The measure comprises three items, for example: “*In my department, we usually help each other*.” Confirmatory factor analysis showed a one-factor structure, with acceptable item loadings above 0.40 and acceptable fit. Cronbach's alpha and omega for the scale in the present study were 0.88 [0.87, 0.88] and 0.88 [0.88, 0.88], indicating good reliability.

#### Group-Level Measures

##### TEI

Team emotional intelligence was assessed using the Team-Trait Meta Mood Scale (T-TMMS; Aritzeta et al., 2020). The T-TMMS is a self-report questionnaire that measures: (1) the degree to which leaders of the same team consider that their team (reference group) pays attention to and values the feelings of teammates, (2) whether there is clarity rather than confusion about the emotions felt in the team, and (3) whether positive thinking is used to repair negative moods in the team. The Cronbach's alphas and omegas for the three dimensions (three items each) of the T-TMMS were 0.76 [0.73, 0.77] and 0.76 [0.74, 0.79] for Attention, 0.80 [0.79, 0.82], and 0.81 [0.79, 0.83] for Clarity, and 0.89 [0.88, 0.90] and 0.89 [0.88, 0.90] for Repair. The overall Cronbach's alpha and omega for the scale were 0.91 [0.91, 0.92] and 0.92 [0.91, 0.92], respectively.

##### Transformational Leadership

The scale used to measure group perception of exercised leadership was adapted from two previously published scales, changing the individual reference point to the group reference point (i.e., changing the reference framework from the individual to the group level) For example: “we have a clear understanding of where we want our unit to be in 5 years.”

Specifically, we adapted Rafferty and Griffin (2006) scale for the Vision, Positive Leadership, and Supportive Leadership dimensions, and the Organizational Culture Inventory (OCI; Cooke and Lafferty, 1983) for the Goal Emphasis dimension.

Confirmatory factor analysis (CFA) was then conducted to confirm the factor structure of the scale. The model showed a good fit (χ^2^df = 227.48, *p* = 0.0001, confirmatory fit index [CFI] = 0.97, Tucker-Lewis index [TLI] = 0.96, root mean square error of approximation [RMSEA] = 0.06, 90%) with adequate factor loadings on four dimensions, thus replicating the structure of the original scale. The Cronbach's alphas for the four dimensions (Vision, Positive Leadership, Supportive Leadership, and Goal Emphasis) were 0.85 [0.85, 0.88], 0.84 [0.83, 0.86], 0.89 [0.86, 0.89], and 0.88 [0.87, 0.89], respectively, and 0.91 [0.91, 0.92] for the total scale. The omegas were 0.87 [0.86, 0.88] for Vision, 0.84 [0.83, 0.86] for Positive Leadership, 0.89 [0.88, 0.90] for Supportive Leadership, 0.88 [0.87, 0.89] for Goal Emphasis, and 0.92 [0.91, 0.92] for the total scale.

## Results

### Descriptive Statistics and Aggregation Indices

In order to examine whether it was appropriate to aggregate individual responses to team-level constructs, we followed the procedure described by Van Mierlo et al. (2009). This includes an examination of the r^*^wg index and two intraclass correlation coefficients, ICC1 and 2. The r^*^wg values are considered as a measure of agreement within the group, ICC1 specifies the proportion of variance in ratings that is due to team membership, and ICC2 specifies the reliability of team mean differences (Klein et al., 2000). Bliese (2000) has stated that ICC1 values exceeding 0.05 can be considered sufficient to warrant aggregation. LeBreton and Senter (2008) suggested that ICC2 values in the range 0.70–0.85 were an appropriate cut-off, and they also recommended that r^*^wg values be interpreted as follows: between 0.51 and 0.70, moderate agreement; between 0.71 and 0.90, strong agreement; and between 0.91 and 1, very strong agreement.

For cohesion, we obtained values between 0.14 and 0.23 for ICC1, between 0.80 and 0.87 for ICC2, and between 0.69 and 0.70 for r^*^wg. For the emotional state “passion,” the values were 0.16 for ICC1, 0.82 for ICC2, and 0.80 for r^*^wg. Thus, we consider that the ICC1, ICC2, and r^*^wg indices justify the aggregation of individual responses.

Descriptive statistics for all variables, including the means, standard deviations, and bivariate correlations between variables, are shown in Table 1.

**Table 1:** Descriptive statistics and correlations.

**Variables**	**Mean (SD) Individual**	**Mean (SD) Group**	**1**	**2**	**3**	**4**	
1. Transformational leadership	–	4.60 (0.48)	1	0.62^**^	0.17^*^	0.10	
2. TEI	–	4.45 (0.50)	–	1	0.22^**^	0.17^**^	Group level (*N* = 188)
3. Passion	4.25 (1.04)	4.34 (0.50)	–	–	1	48^**^	
4. Cohesion	4.18 (1.24)	4.27 (0.61)	–	–	0.35^**^	1	
			Individual level (*N* = 4,564)				

** p < 0.01;

* p < 0.05

### Hypotheses Testing

**Figure 2:**
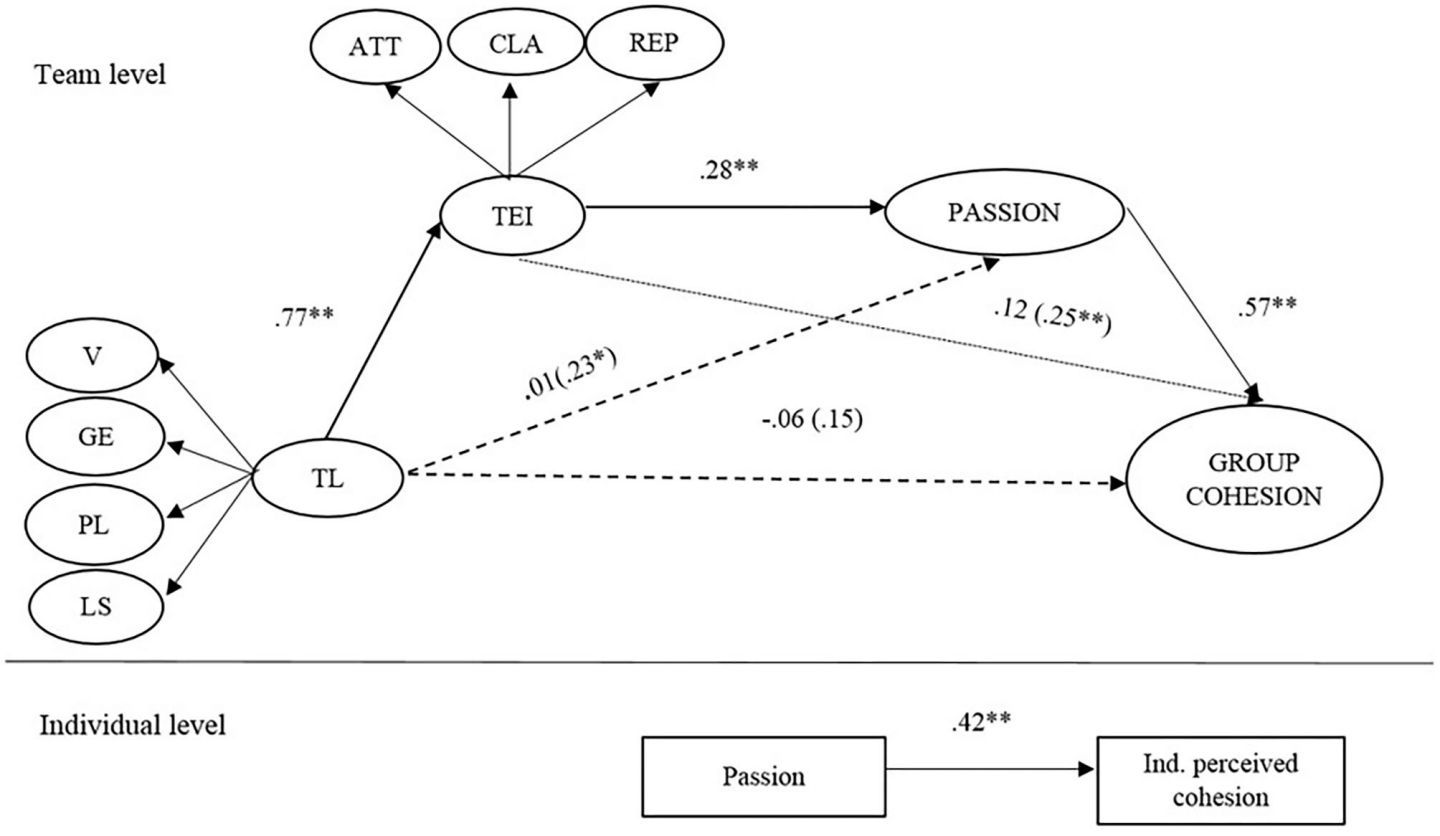
Model results. TL, Transformational leadership; V, Vision; GE, Goal Emphasis; PL, Positive Leadership; SL, Supportive Leadership; TEI, Team emotional intelligence; ATT, Attention; CLA, Clarity; REP, Repair. **p < 0.01; *p < 0.05.

We tested our hypotheses by means of multilevel structural equation modeling with Mplus. The results are presented in Figure 2. The model fit indexes (CFI = 0.96; TLI = 0.93; RMSEA = 0.02; SRMR(W) [standardized root mean square residual for the within-level model] = 0.01; SRMR(B) [standardized root mean square residual for the between-level model] = 0.05) indicated good fit of the analyzed model. The effect of the size of the organizational area was controlled for in the model, and as none of the relationships for this control variable were significant (β_*TEI*_ = −0.01, *n.s*.; β_*Passion*_ = 0.01, *n.s*.; β_*Cohesion*_ = 0.01, *n.s*.) the paths were eliminated in Figure 2.

In support of Hypothesis 1, and after controlling for the effect of the area size, transformational leadership was positively related to TEI at the group level (β = 0.77, *p* < 0.01). Hypothesis 5 was supported as TEI was positively related to passion (β = 0.28, *p* < 0.01). Hypothesis 4 was also supported, since at the individual level, passion was positively related to perceived cohesion (β = 0.42, *p* < 0.01), while at the group level, passion was positively related to group cohesion (β = 0.57, *p* < 0.01).

The overall model proposes that both TEI and the emotional state “passion” mediate the relationship between transformational leadership and cohesion at the group level. No significant effects were found in the direct relationship between transformational leadership of management teams and group cohesion prior to introducing the two mediators, therefore hypothesis 3 was not supported. However, TEI was found to mediate the relationship between transformational leadership and passion, since the direct effect of transformational leadership on passion changed from significant (β = 0.23, *p* < 0.05), supporting hypothesis 2, to not significant (β = 0.01, *n.s*.), indicating a full mediation effect. In addition, passion fully mediated the relationship between TEI and group cohesion (effect before mediation: β = 0.25, *p* < 0.01; effect after mediation: β = 0.12, *n.s*.). The indirect effect of transformational leadership through TEI and passion was calculated using the model constraint function of Mplus. The results showed a significant indirect effect of transformational leadership on cohesion (via TEI and passion) (*indirect* = *0.11, p* < 0.01).

We then compared this model with several alternative models at the team level. The results are presented in Table 2. Model 1 is the full mediation model, while in model 2 we added the direct paths from transformational leadership to passion and from TEI to cohesion. Models 3 and 4 were tested to investigate the effects of changing the order of variables. The fit statistics of these models were worse and some paths were non-significant in model 3. Therefore, we conclude that model 1 was the best model for the team level.

**Table 2:** Alternative models.

**Model and structure**	***x*^2^**	***df***	**RMSEA**	**CFI**	**SRMR**
TL → TEI → PAS → COH	17.81	14	0.038	0.99	0.03
TL → TEI → PAS → COH and TL → PAS and TEI → COH	16.03	12	0.042	0.99	0.03
TEI → TL → PAS → COH	19.75	14	0.047	0.98	0.04
TL → TEI → COH → PAS	22.44	14	0.057	0.98	0.06

*TL, Transformational leadership; TEI, Team emotional intelligence; PAS, Passion; COH, Cohesion*.

## Discussion

The present study sought to shed light on the emotional mechanisms that underpin the relationship between the transformational leadership of management teams and cohesion among workers, and also to analyze the role played by TEI in this relationship. Our findings overall are consistent with previous studies suggesting that teams with high levels of TEI are better able to understand the functioning of their organization and what this implies in terms of managing emotions (Stubbs and Wolff, 2008). More specifically, the results provide support for our predictions. First, transformational leadership behaviors are positively associated with higher levels of TEI in management teams (H1) and higher passion (H2). Second, the TEI of leader teams fully mediates the relationship between transformational leadership behaviors and the positive emotional or affective state of passion of subordinates at the team level (H5). Third, the high-intensity positive emotions we referred to as the affective state of “passion” mediate the relationship between TEI and subordinates' cohesion (H4.b). And fourth, at the individual level, subordinates' passion influences their perception of cohesion (H4.a).

Analysis of our overall model suggests that management teams composed of transformational leaders have higher TEI and generate more positive emotions in their followers, who then experience greater cohesion within the team. Following Druskat and Wolff (2001), we consider that transformational leadership helps to generate emotionally intelligent norms and patterns of behavior which enable the team to work more efficiently. Thus, teams high in TEI are able to generate norms for adequately managing conflicts that arise within the group (Ayoco et al., 2008), which in turn promotes greater cooperation, coordination, and communication among members (Lee and Wong, 2019).

The mediation effects we identified are in line with the results of previous studies suggesting that teams with high TEI are better at recognizing and responding to the emotions and needs of their followers (George, 2000; Chang et al., 2011). Team members' interpersonal relationships may influence employees' individual affect through trickle-down effects and emotional contagion, leading to an organizational management response to the leaders (Tse et al., 2018). In this respect, one might consider that teams high in TEI are able to generate more positive emotions among subordinates, for whom they constitute a positive affective event (Weiss and Cropanzano, 1996; Weiss, 2002).

The present study also proposed that the effect which leaders have on workers' cohesion could be due, in part, to an increase in high-intensity positive emotions. Although the relationship between the emotional state of passion and workers' cohesion has recently been demonstrated (Diener et al., 2020), the mediation effect we found here further highlights the considerable importance that positive emotions may have in the relationship between leaders and subordinates.

Although some previous studies have linked transformational leadership to cohesion (Sahib and Wilderom, 2017), we surprisingly did not find a significant direct effect between these two constructs. This may be because, unlike previous studies, we examined the relationship between the management team and employees from a multilevel perspective. According to Wo et al. (2019), cognition-based constructs such as leadership may be harder to transmit across different levels of an organizational hierarchy than are affect-based constructs, which could explain the lack of a significant effect of transformational leadership on cohesion.

As noted in the introduction, there has been little research on organizational leadership style's effect on workers responses and well-being, and the emotional mechanisms that underpin this relationship. Our study contributes to the literature focused on the organizational level by highlighting the role of emotions in the relationship between management teams' behaviors and employees' cohesion. More specifically, our results show that understanding and managing emotions is a central part of leadership effectiveness. In doing so, the present study contributes to understanding why transformational leadership behaviors affect well-being at work and what the keys to develop an effective leadership are.

Analyzing the aforementioned variables in a single study is important because it contributes to the theoretical domains of group affect and leadership. In this respect, our study sheds light on the question of how and why transformational leadership and TEI may enhance well-being (Colquitt and Zapata-Phelan, 2007). Moreover, although several studies have demonstrated the effect of individual EI on different organizational variables (Miao et al., 2016), only a few studies have analyzed the corresponding group-level construct, that is, TEI. Here we integrated the individual and team level in emotion research (Ashkanasy and Dorris, 2017) by examining emotions and their influence on cohesion from a multilevel perspective. It should also be noted that we considered perceptions from two different sources, namely subordinates and leaders, thus adding to knowledge of leader-member exchange processes (Tse et al., 2018).

### Practical Implications

Our findings have a number of implications. First, they highlight the importance of emotions and affectivity at both the individual and team levels, thus underlining why managers need to consider TEI as an important skill when training project teams. In this respect, our results could be used to promote workers' well-being and create emotionally healthier organizations. For instance, activities aimed at increasing leader teams' emotional intelligence would indirectly impact the well-being of workers and, ultimately, of the organization. In this context, recent research on large projects has found that training can improve EI in project team members (Kotsou et al., 2018).

The use of the multilevel theoretical framework, rather than focusing solely on the shared perception of workers, helps to advance on the team-based EI research by defining the relations between TEI and workers' responses. This research challenges and maybe complements the classical view of the relationship between leadership and employees' outcomes, underlining the importance of both team-level and affective variables for these responses. Team emotional intelligence and the affective responses of employees combine to create structural configurations that influence working processes, shaping the linkages between leadership and cohesion.

Finally, it should be mentioned that some researchers (e.g., Ashkanasy, 2003; Troth et al., 2012) have called for the development of models examining the effect of EI on performance at the team level, including identification of the mechanisms through which TEI may impact outcomes at work. The model developed in this study shows how TEI mediates the relationship between leadership and team cohesion. Our findings therefore add to knowledge about team cohesion by providing an explanation of how TEI and passion mediate the association between leadership and cohesion.

### Limitations and Future Directions

Our study has certain limitations that need to be considered. First, the results are based on self-report data and it is possible that they are affected by social desirability bias. In addition, even if our data were collected from two different sources, the results still can present common method bias in the relationship we established from the same sources (Transformational leadership to TEI and Passion to Cohesion). Future studies should therefore employ more objective measures to verify the impact of TEI in organizations and avoid common method bias. It would also be useful to examine EI and its relationship to performance in different cultural contexts and different kinds of projects. In this respect, the fact that we examined the hypothesized relationships within a single organizational context limits the generalizability of the findings. A related issue to consider here is that all the organizations included in this study were cooperatives, whose characteristics and functioning differ considerably from other types of company. Future studies should therefore explore the observed relationships in different organizational contexts.

A further limitation to note is that our study does not capture the dynamic nature of EI in the workplace because we did not collect longitudinal or qualitative data. Consequently, conclusions about causality cannot be drawn from our results. In addition, we only considered emotions classified as high-intensity positive emotions, those which have been shown in the literature to have a greater effect. A task for future research would therefore be to investigate the impact of other types of emotion on the process of leadership.

Finally, it is worth mentioning that we did not examine gender differences in transformational leadership, which may be relevant since the leadership teams in our sample were not homogeneous in this respect. About 30% of teams were comprised solely of men, while the remainder had one or more female members; there were no women-only leadership teams. In light of recent findings in this context (Hackett et al., 2018), future studies should examine whether the gender composition of teams may influence the mediation effect observed here.

Despite these limitations, our study provides empirical results and adds to knowledge about the influence of emotions on organizations and effective leadership. More specifically, it highlights the need for organizations to focus not only on promoting transformational leadership styles within their management teams but also on the development of emotional skills such as TEI that can help teams to function better and be capable of recognizing and meeting the needs of workers. In other words, that they achieve effective leadership which enables them to become healthy as well as productive organizations.

## Data Availability Statement

The raw data supporting the conclusions of this article will be made available by the authors, without undue reservation.

## Ethics Statement

The studies involving human participants were reviewed and approved by University of Mondragón. The patients/participants provided their written informed consent to participate in this study.

## Author Contributions

RM together with AA and EM-M were responsible of developing the theoretical foundations of the manuscript (introduction, discussion, and conclusion). AG together with RM and AA were responsible of the methodological part of the manuscript and especially of the statistical analysis. UE was responsible of the process for gathering data and reviewing the manuscript. GS contributed to the theoretical development and review process. All authors contributed to the article and approved the submitted version.

## Conflict of Interest

The authors declare that the research was conducted in the absence of any commercial or financial relationships that could be construed as a potential conflict of interest.
